# Acute TSH stimulation in vivo does not alter serum PCSK9 levels

**DOI:** 10.1186/1756-6614-7-4

**Published:** 2014-05-01

**Authors:** AnneMarie Gagnon, Moeber Mahzari, Heather A Lochnan, Alexander Sorisky

**Affiliations:** 1 Chronic Disease Program, Ottawa Hospital Research Institute, University of Ottawa, Ottawa, Canada; 2 Department of Medicine, University of Ottawa, Ottawa, Canada; 3 Department Biochemistry, Microbiology &Immunology, University of Ottawa, Ottawa, Canada; 4 Ottawa Hospital Research Institute, 501 Smyth Road, Ottawa, Ontario K1H 8L6, Canada

**Keywords:** TSH, PCSK9, Thyroid, Liver

## Abstract

**Background:**

It is now recognized that TSH can act on targets other than the thyroid, including the liver. Elevated serum TSH levels in euthyroid subjects were recently reported to correlate with high values of serum proprotein convertase subtilisin/kexin type 9 (PCSK9). This protein, expressed and secreted by hepatocytes, promotes higher LDL-cholesterol levels. We tested whether an acute increase of TSH levels following administration of TSH in vivo would raise PCSK9 levels in patients who had previously undergone total thyroidectomy and radioablation for thyroid cancer.

**Findings:**

TSH levels rose from 0.64 ± 1.02 mU/L on day 1 to 98.66 ± 4.83 mU/L on day 3, following injections of recombinant human TSH (on days 1 and 2). PCSK9 levels were 330 ± 99 ng/ml on day 1, and did not change on days 3 or 5 in response to TSH stimulation.

**Conclusions:**

Although a positive correlation between TSH and PCSK9 in euthyroid subjects has raised the possibility that TSH might act on the liver to raise PCSK9 values, our data show that PCSK9 levels are not affected by acute elevations of TSH levels. Whether chronic elevations of TSH are needed to upregulate PCSK9 remains to be determined.

## Findings

### Introduction

The TSH receptor is classically expressed in thyrocytes. In response to TSH, it activates downstream intracellular signaling events to stimulate thyroid hormone production and cell proliferation [1]. It is now appreciated that TSH receptors are also expressed in non-thyroidal tissues [2]. The physiological significance of this extra-thyroidal expression has not yet been clarified, but regulation of bone development, adipose tissue remodelling, and hepatocyte cholesterol metabolism have been proposed [3-5].

Acute non-thyroidal effects of TSH, in the setting of normal thyroid hormone levels, can be studied in vivo with recombinant human (rh)TSH stimulation. It is used for thyroid cancer screening in patients who have undergone total thyroidectomy followed by radioablation of any remnant tissue. For example, rhTSH stimulation has been shown to increase circulating IL-6, CRP, oxidative stress markers, and non-esterified fatty acids, and to decrease endothelial relaxation in these patients who have no thyroid glands [6-8].

Levels of TSH, but not free T4 (FT4), correlate positively with proprotein convertase subtilisin/kexin type 9 (PCSK9) in euthyroid, non-obese subjects [9]. PCSK9, expressed in hepatocytes, is secreted and binds to the LDL receptor. It directs the receptor to lysosomal degradation rather than a recycling pathway, lowering the number of LDL receptors expressed on the cell surface, and thereby leading to an increase in LDL cholesterol [10] . Hepatocytes are an extra-thyroidal target of TSH, which regulates the expression of 3-hydroxy-3-methyl-glutaryl coenzyme A reductase, and therefore, cholesterol metabolism [5]. Therefore, it was suggested that TSH might possibly act on hepatocytes to raise PCSK9 levels [9].

Our objective was to determine if PCSK9 levels would rise in patients previously treated for thyroid cancer undergoing acute rhTSH stimulation.

## Patients and methods

### Patients

**Figure 1:**
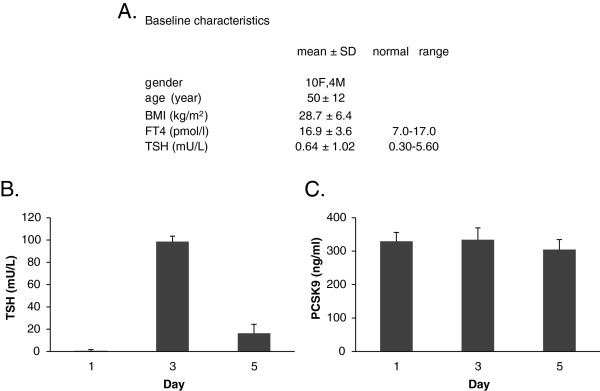
**Acute rhTSH administration does not alter serum PCSK9 levels.** Baseline characteristics of the patient population and normal laboratory range values **(A)**. Serum TSH **(B)** and PCSK9 **(C)** levels at baseline (day 1), on day 3 and day 5 after rhTSH injection. Results are the mean ± SD of 14 patient samples.

14 patients (10 women) were recruited (Ottawa Hospital Research Ethics Board #2006558). All had undergone total thyroidectomy and radioablative iodine therapy for thyroid cancer, and were on L-thyroxine treatment. They were healthy without evidence of metastatic disease. Each received 0.9 mg rhTSH on days 1 and 2, and blood samples were drawn on days 1 (prior to rhTSH), 3, and 5. Figure 1A shows the baseline characteristics.

### Assays

PCSK9 was measured using the PCSK9 Quantikine ELISA kit (#DPC900; R&D Systems). TSH and free thyroxine were measured in The Ottawa Hospital clinical biochemistry laboratory by AutoIA (Abbot Dxl).

### Statistical analysis

Data were assessed by analysis of variance, and by posthoc Newman-Keul tests for multiple comparisons, or by unpaired t-test, with P < 0.05 taken as significant. Values of TSH reported by the clinical laboratory as “>100 mU/L” were taken as “100 mU/L” for calculation of mean ± SD. Pearson’s regression and correlation analysis were performed using Graph-Pad Instat version 3.

## Results

### Basal studies

Overall basal parameters are shown in Figure 1A. At baseline (day 1), the value of PCSK9 (mean ± SD) in all participants was 330 ± 99 ng/ml. In the females (n = 10), baseline value was 351 ± 90 ng/ml, and in the males (n = 4), it was 276 ± 113 ng/ml. The difference between PCSK9 values in females versus males was not significant.

There was a trend towards a correlation between baseline PCSK9 values in all participants and BMI (r = 0.47; p =0.09). Further analysis showed this was a result of a significant positive correlation with BMI in the females (r = 0.783, p = 0.0073).

In patients with BMI < 30 (n = 10), PCSK9 (mean ± SD) was 317 ± 83 ng/ml versus 360 ± 141 ng/ml in patients with BMI > 30 (n = 4) (not significant).

There was no correlation between baseline PCSK9 and age, TSH, or FT4 value.

#### Stimulated studies

Administration of rhTSH increased TSH levels (mean ± SD) from 0.64 ± 1.02 mU/L on day 1, to 98.66 ± 4.83 mU/L on day 3, and to 16.43 ± 7.76 mU/L on day 5 (Figure 1B). There was no change in PCSK9 values from the baseline value (Figure 1C). PCSK9 (mean ± SD) was 334 ± 139 ng/ml on day 3, and 305 ± 111 ng/ml on day 5. The same lack of response was also observed when participants were divided according to gender or obesity status (BMI <30 versus > 30).

## Conclusions

PCSK9, an important regulator of lipoprotein metabolism [10], was recently reported to correlate positively with TSH levels in non-obese euthyroid subjects [9]. Hepatocytes, the major source of PCSK9, are TSH-responsive cells with respect to 3-hydroxy-3-methyl-glutaryl coenzyme A reductase expression and cholesterol metabolism [5]. On that basis, it was proposed that TSH might act on hepatocytes to stimulate PCSK9 release [9]. This prompted us to determine whether acute rhTSH administration would raise PCSK9 levels.

Our results indicate there is no change of serum levels of PCSK9 in response to acute rhTSH stimulation in non-obese or obese patients, despite the high levels of TSH achieved. Our baseline PCSK9 data, pointing to a positive correlation with BMI, are in agreement with other studies [10] .

Our observations are not consistent with the recently reported correlation of PCSK9 and TSH in 64 non-obese euthyroid subjects [9]. There was no such correlation of PCSK9 with FT4, so that effects on PCSK9 were thought to be due to direct effects of TSH on the hepatocyte independent of hypothyroidism.

However, several limitations of our study should be considered. Our study sample is relatively small, and may have been under-powered to detect a small response of PCSK9 to rhTSH. The stimulation protocol with rhTSH in our patients is acute, and perhaps a chronic elevation of TSH over weeks to months is required to alter hepatocyte PCSK9 production and secretion to elevate serum values. We were also limited to sampling blood on day 3 and day 5, according to the clinical protocol, so we cannot rule out either a very early response to rhTSH, eg on day 2, or a delayed response after day5. We also note that thyroxine therapy in our patients is aimed at keeping FT4 values toward the high end of normal, in order to target endogenous TSH levels to the low end of normal, and it is possible that might attenuate responsiveness to exogenous rhTSH administration.

Further studies in patients with subclinical hypothyroidism, characterized by chronically elevated TSH but normal thyroxine levels, may clarify whether TSH is positively correlated with PCSK9. It may also be informative to treat human hepatocytes in culture with TSH to determine if it can directly increase PCSK9 expression and/or secretion.

## Competing interests

The authors declare they have no competing interests.

## Authors’ contributions

AG carried out the PCSK9 assays, and participated in the study design and analysis of data. MM participated in sample processing and data analysis. HAL helped with study design. AS conceived of the study design and wrote the manuscript with AG. All authors read and approved the final manuscript.
